# Influence of *Fusobacterium nucleatum* on Tumor Necrosis Factor Alpha Therapy in Ulcerative Colitis

**DOI:** 10.1155/mi/5035877

**Published:** 2025-10-31

**Authors:** Bilge Şenyüz, Nalan Gülşen Ünal, Başak Doğanavşargil Yakut, Ataç Uzel, Cumhur Gündüz, Sunde Yılmaz Süslüer

**Affiliations:** ^1^Department of Medical Biology, Ege University Faculty of Medicine, Izmir, Türkiye; ^2^Department of Gastroenterology, Ege University Faculty of Medicine, Izmir, Türkiye; ^3^Department of Pathology, Ege University Faculty of Medicine, Izmir, Türkiye; ^4^Department of Microbiology, Ege University Faculty of Science, Izmir, Türkiye

**Keywords:** anti-TNF therapy, *Fusobacterium nucleatum*, microbiota dysbiosis, TNF-α, ulcerative colitis

## Abstract

Inflammatory bowel disease (IBD), including ulcerative colitis (UC), involves chronic gastrointestinal inflammation, with tumor necrosis factor alpha (TNF-α) playing a key role. Anti-TNF therapy is widely used, but not all UC patients respond, suggesting additional contributing factors. Gut microbiota alterations, particularly dysbiosis, may influence treatment outcomes. This study examines the relationship between *Fusobacterium nucleatum* (*F. nucleatum*) density and TNF-α expression in UC patients receiving anti-TNF therapy. Biopsy samples from responders (*n* = 10), nonresponders (*n* = 10), and healthy controls (*n* = 10) were analyzed using real-time PCR. *Fusobacterium nucleatum* density was significantly higher in nonresponders than in responders (3.2-fold, *p*  < 0.05) and controls (fivefold, *p*  < 0.05). TNF-α expression was elevated in both UC groups. These findings suggest *F. nucleatum* may contribute to anti-TNF therapy resistance by modulating intestinal inflammation, highlighting its potential as a biomarker for treatment prediction.

## 1. Introduction

Inflammatory bowel disease (IBD) refers to a group of disorders characterized by chronic inflammation of the gastrointestinal tract [1]. While factors such as genetic predisposition, diet, smoking, and alterations in the microbiota are known to contribute to disease development, the precise etiology remains unclear [2, 3].

Ulcerative colitis (UC), one of the primary forms of IBD, is an autoimmune condition wherein the immune system attacks the intestinal lining, leading to mucosal damage and ulceration [4]. UC is distinguished by mucosal inflammation that originates in the rectum and extends proximally through the colon, resulting in superficial intestinal wall injury [5, 6]. The primary goals of UC treatment are to achieve rapid remission, enhance the quality of life, reduce long-term steroid dependance, and minimize both the disease's and the treatment's adverse effects [7].

The balance between proinflammatory and anti-inflammatory cytokines plays a crucial role in the pathogenesis of IBD.

Tumor necrosis factor (TNF), a pivotal cytokine, functions as a cell signaling protein involved in orchestrating the inflammatory response and regulating immune cell activity. It is primarily produced by macrophages [8] in response to stimuli, such as Gram-negative and Gram-positive bacteria, lipopolysaccharides, and cytokines like IL-2, exerting its effects through receptor binding [9]. TNF-α, in particular, plays a central role in the immunopathogenesis of IBD. TNF produced within the lamina propria has been shown to significantly compromise intestinal barrier integrity and also elevated levels of TNF-α are associated with decreased intestinal barrier function in UC. It is possible to prevent disease complications by changing the course of the disease. Current therapeutic strategies for UC, particularly in severe cases, aim to reduce inflammation via anti-inflammatory agents, immunosuppressants, corticosteroids, and biologic agents targeting TNF-α [10]. Anti-TNF agents, monoclonal antibodies engineered to neutralize TNF, have demonstrated efficacy in both inducing and maintaining remission in UC patients [11, 12].

The human intestinal microbiota consists of approximately 100 trillion microorganisms, including bacteria, fungi, viruses, and other entities [13]. Alterations in the composition and function (dysbiosis) of microbiota are being linked to various diseases [14]. While the pathogenesis of IBD remains incompletely understood, there is growing evidence implicating dysbiosis in disease progression [15, 16]. Studies show reduced bacterial diversity and alterations in microbial composition in UC patients compared to healthy individuals, especially a decrease in bacteria with anti-inflammatory properties and an increase in bacteria with pro-inflammatory characteristics [17, 18].

Among 500–1000 different bacterial species, the dominant bacterial phyla in the human gut are Firmicutes, Bacteroidetes, Proteobacteria, and Actinobacteria [19]. With shifts toward pathogenic species like *Klebsiella pneumoniae*, *Enterococcus faecalis*, and *Escherichia coli* observed in IBD patients [20].

Among these, *Fusobacterium nucleatum* (*F. nucleatum*) is a Gram-negative anaerobic bacterium typically found in the oral cavity and gastrointestinal tract exhibiting variable pathogenic potential. Although *F. nucleatum* has been implicated in in various intestinal diseases, its role in UC pathogenesis remains unclear [21, 22]. It is known to interact with E-cadherin and activate several pathways, such as TLR4/MYD88, NF-κB, Wnt, and autophagy, promoting inflammation and tumor progression [23, 24]. However, the specific involvement of *F. nucleatum* in UC has yet to be fully elucidated [22]. The aim of this study is to investigate whether there is a relationship between Fusobacterium nucletaum level and primary response to anti-TNF-α treatment in UC patients.

## 2. Materials and Methods

The study was conducted using biopsy samples from patients treated at the Gastroenterology Department of Ege University Faculty of Medicine. Patients were categorized based on their response to anti-TNF-α therapy: responders (*n* = 10), nonresponders (*n* = 10), and healthy controls (*n* = 10). Formalin-fixed, paraffin-embedded (FFPE) tissues were used for subsequent analyses.

### 2.1. Patient Selection

Patients were selected from those registered and under follow-up in the IBD database of the Ege University Gastroenterology Clinic. The study included patients who had been diagnosed with UC at least 6 months earlier based on clinical, laboratory, endoscopic, and histopathological criteria, who were unresponsive/intolerant to conventional therapy, or who developed side effects, and for whom anti-TNF-α therapy was initiated.

Patients presenting with active clinical and laboratory symptoms and findings, but without endoscopic activity, were excluded from the study. Demographic and disease characteristics of patients were obtained from the electronic medical records.

Before the initiation of anti-TNF -α therapy, colonoscopic, or rectosigmoidoscopic evaluations were made to ensure endoscopic activity of UC. Endoscopic activity was assessed according to the Mayo endoscopic subscore (MES). Patients with an MES of 0 or 1 were classified as “disease in remission” and excluded, whereas those with an MES of 2 or 3 were classified as “active disease” and included in the study [25–28].

Patients were divided into two groups based on their clinical response to anti-TNF-α therapy at month 3. The anti-TNF-α therapy response was evaluated according to the total partial Mayo score (TPMS) and C-reactive protein (CRP) level at the third month of treatment. Patients with a TPMS > 4 and CRP > 5 mg/L were interpreted as having active disease and classified as group “primary nonresponders”, whereas those with a TPMS ≤ 4 and CRP <5 mg/L were considered the “primary responders” group. The control group comprised patients over 50 years of age who underwent screening colonoscopy for colorectal cancer and had no pathological findings. All biopsies were obtained from the rectum before the initiation of anti-TNF-α therapy.

### 2.2. DNA and RNA Extraction

DNA and RNA were extracted from 8 µm × 5 µm sections of FFPE biopsy samples from both patient and control groups using the “QIAamp DNA FFPE Tissue Kit” and “RNeasy FFPE Kit” (Qiagen), respectively, according to the manufacturer's protocols.

### 2.3. Quantification and Purity Assessment of DNA and RNA

The concentration and purity of the extracted DNA and RNA were assessed by measuring absorbance at 260/280 nm and 230/260 nm using a Nanodrop instrument (Thermo Scientific, USA). Only RNA samples with A260/A280 and A230/A260 absorbance ratios exceeding 2.0 were deemed suitable for further analysis.

### 2.4. Complementary DNA (cDNA) Synthesis

cDNA synthesis from total RNA was performed using the “QuantiTect Reverse Transcription Kit” (Qiagen), following the manufacturer's instructions.

### 2.5. PCR Analysis

The abundance of *F. nucleatum* was determined by real-time PCR, while TNF-α mRNA expression levels were analyzed via qRT-PCR using specific primers (Table 1) and EnTurbo SYBR Green PCR SuperMix (ELK Biotechnology) on a LightCycler 480 II instrument (Roche Life Science). Gene expression was normalized to the average expression of the reference genes RNU6 and GAPDH, with relative expression determined using the 2^−ΔΔCT^ method.

### 2.6. Statistical Analysis

Standard curve and melting curve analyses were performed across four logarithmic phases to assess PCR efficiency. Gene expression profiles were analyzed using the 2^−ΔΔCT^ method. The statistical relationship between *F. nucleatum* and TNF-α expression was analyzed using the web-based Qiagen GeneGlobe Data Analysis Center (GeneGlobe Data Analysis Center | NGS and PCR Analysis Tools | GeneGlobe, n.d.). Comparisons between groups were made using Student's *t*-test, with a *p*-value < 0.05 considered statistically significant.

## 3. Results

### 3.1. Patient Demographics

The mean age of the participants was 55.3 years (6F, 4M) in the control group, 39.6 years (2F, 8M) in the anti-TNF-α responders, and 43.6 years (4F, 6M) in the nonresponders. Demographic and disease characteristics of patients were detailed in Table 2. All of the UC patients had endoscopic activity (EMS was 2 or 3 points) at the time of the colonoscopic or rectosigmoidoscopic procedure before anti-TNF therapy. No statistically significant differences were observed between the patient groups in terms of demographic or disease characteristics (*p*  > 0.001). A statistically significant difference was found only in the mean age between the patient groups and the control groups (*p*  < 0.001) (Table 2.)

### 3.2. *Fusobacterium nucleatum* Density

**Figure 1:**
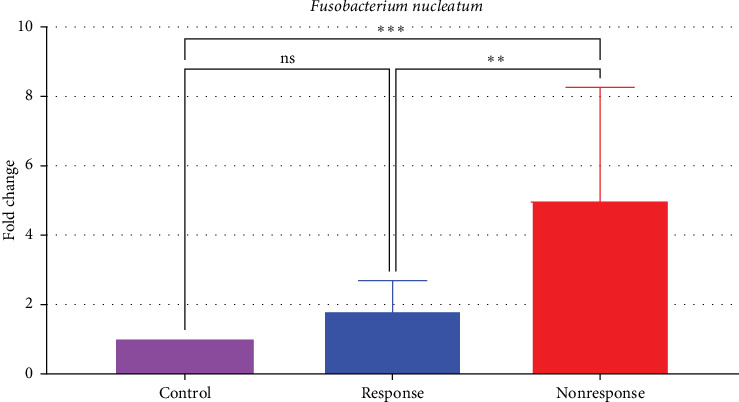
Comparison of *F. nucleatum* DNA amounts in the anti-TNF-α treatment responsive, nonresponsive and control groups (*⁣*^*∗∗∗*^: 0.0002, *⁣*^*∗∗*^: 0.0028; ns, nonsignificant).

*Fusobacterium nucleatum* density was significantly higher in nonresponders compared to the control group, with a fivefold increase (*p*  < 0.05). In responders, the density was 1.8 times higher than in controls, though this difference was not statistically significant (*p*  > 0.05). When comparing responders to nonresponders, *F. nucleatum* density was significantly 3.2 times higher in the nonresponders (*p*  < 0.05) (Table 3, Figure 1).

### 3.3. TNF-α mRNA Expression Levels

**Figure 2:**
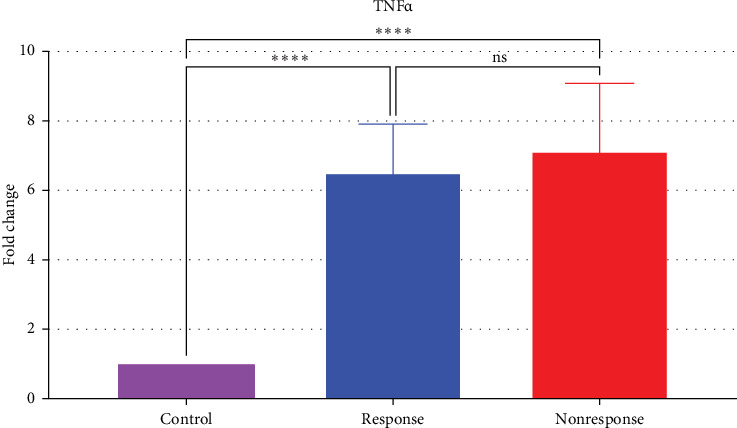
Comparison of TNF-α mRNA expression levels of the TNF-α responsive, nonresponsive and control groups (*⁣*^*∗∗∗∗*^, significant; ns, nonsignificant).

Both responder and nonresponder groups exhibited significantly elevated TNF-α mRNA levels compared to controls (sixfold and sevenfold, respectively, *p*  < 0.05). There was no significant difference in TNF-α mRNA levels between responders and nonresponders (*p*  > 0.05) (Table 4, Figure 2).

## 4. Discussion

The etiology of IBD, including UC, remains poorly understood, but it is influenced by the presence of normal intestinal microflora and potentially unidentified pathogens.

Recent evidence indicates a link between gut microbiota and immune-mediated gastrointestinal diseases, such as UC. However, the mechanism by which *F. nucleatum* induces intestinal epithelial cell death in UC remains unclear.

Su et al. [29] investigated the role of *F. nucleatum* in intestinal epithelial cell death and its exacerbation of UC by using *F. nucleatum* lipopolysaccharide. Their findings revealed that *F. nucleatum* was abundant in UC tissues and correlated with clinical features. They demonstrated that *F. nucleatum* worsened epithelial cell death by promoting autophagy, and that this effect could be inhibited by autophagy blockers such as chloroquine, 3-methyladenine, and Atg5 silencing, suggesting that *F. nucleatum* may contribute to UC through excessive activation of autophagy in intestinal epithelial cells [29].

In a complementary study, Lin et al. [22] developed experimental models by administering dextran sulfate sodium (DSS) solution and *F. nucleatum* to mice. They monitored survival rates, weight changes, and disease activity, while assessing the density of *F. nucleatum* in the colon along with inflammatory cytokine levels. Their findings indicated that *F. nucleatum* facilitated alveolar bone loss and specifically colonized infected colon tissue. Mice treated with DSS exhibited destruction of intestinal architecture, increased expression of interleukin-1 beta (IL-1β) and TNF-α, decreased IL-10 levels, heightened apoptosis in intestinal epithelial cells, and microbiota dysbiosis. They concluded that *F. nucleatum* exacerbates UC by promoting intestinal inflammation, disrupting the epithelial barrier, and causing microbiota dysbiosis and metabolic disorders [22].

Yamashita et al. [30] investigated the impact of *F. nucleatum* on the remission of DSS-induced colitis in female mice. Mice were divided into a DSS control (DC) group and a DSS + *F. nucleatum* (DF) group, with the DF group receiving oral *F. nucleatum* and both groups given DSS in their drinking water to induce colitis. In Experiment 1, DF mice had significantly higher disease activity indexes (DAIs) (*p*  < 0.05) than DC mice during the healing and second inflammation stages, indicating delayed recovery. In Experiment 2, microbiota analysis showed significantly higher Bacteroides and lower Lachnospiraceae levels in the DF group (*p*  < 0.05). These results suggest that *F. nucleatum* may impair colitis healing by altering gut microbiota.

In another study using DSS-induced colitis in mice and Caco-2 cell lines to elucidate the pathogenicity of *F. nucleatum*, UC was significantly exacerbated in mice that received both DSS and *F. nucleatum*. Furthermore, *F. nucleatum* triggered the release of TNF-α, interferon-γ, IL-1β, IL-6, and IL-17. The authors concluded that *F. nucleatum* may cause intestinal barrier damage and induce abnormal inflammation that exacerbates UC [31].

Murch et al. [32] examined TNF-α serum levels in children with chronic IBD and a significant increase in TNF-α was observed exclusively in children with UC and relapsed Crohn's disease.

Dharmani et al. [33] demonstrated that highly invasive *F. nucleatum* isolates obtained from the inflamed intestines of Crohn's disease patients significantly increased *MUC2* and *TNF-α* gene expression in human colonic epithelial cells and a rat intestinal infection model compared to less invasive strains derived from noninflamed intestines. Notably, only live *F. nucleatum* was capable of stimulating mucus secretion and TNF-α expression upon invading colon cells. In rat colons, mucus secretion increased in response to a highly invasive *F. nucleatum* isolate, whereas it remained unaffected by a low-invasive strain. This suggests that *F. nucleatum* may be a significant pathogen in the etiology of intestinal inflammatory diseases [33].

These studies indicate that *F. nucleatum* increases TNF-α expression. However, the relationship between *F. nucleatum* and TNF-α, along with the response of patients to anti-TNF treatment in UC, has not been thoroughly investigated.

In our study, we examined tissues from patients with UC both before and after anti-TNF treatment, analyzing their response to therapy. We assessed *F. nucleatum* density in the tissues and analyzed TNF-α mRNA expression levels.

Our study demonstrates that *F. nucleatum* density is increased in UC patients, particularly those who do not respond to anti-TNF therapy. These findings suggest that *F. nucleatum* may play a role in modulating patient response to anti-TNF treatment by influencing TNF-α expression and intestinal inflammation.

The mean age in the control group is much higher (55.3 years) comparing (39.6 years in primary responder group and 43.3 years in primary nonresponder group) in the anti-TNF-α treated groups of UC patients. This may be attributable to the fact that, as a tertiary center for UC patients, younger, and refractory cases are more likely to be referred to our clinic, resulting in a younger patient cohort, whereas the control group consisted of individuals over 50 years of age undergoing colorectal cancer screening. The proportion of females is different in the control group comparing to the treated group of patients. The relatively small sample size and the lack of age and sex matching between the control and patient groups are limiations of this study. Further studies with larger sample sizes and matched groups are needed to better elucidate the relationship between *F. nucleatum* and the response to anti-TNF therapy.

## 5. Conclusion

Previous studies have established that *F. nucleatum* plays a crucial role in increasing TNF-α expression. However, the response to anti-TNF treatment has not been extensively analyzed to clarify the relationship between *F. nucleatum* and TNF-α in UC patients. In our study, we analyzed tissues obtained from UC patients prior to anti-TNF treatment. Following treatment initiation, we monitored the patients' responses. We assessed *F. nucleatum* density and examined TNF-α mRNA expression levels. As anticipated, we found that *F. nucleatum* levels increased in patients with UC compared to the control group. Additionally, *F. nucleatum* density was higher in patients who did not respond to anti-TNF treatment than in those who did. Notably, TNF-α mRNA expression levels were significantly elevated in both patient groups—those who responded and those who did not—compared to the control group. Our findings suggest that *F. nucleatum* density may correlate with the response to anti-TNF treatment.

Our results confirm that *F. nucleatum* density is elevated in UC patients, particularly those unresponsive to anti-TNF-α therapy. This suggests a potential link between *F. nucleatum* and treatment resistance, warranting further investigation into its role in UC pathogenesis and therapeutic outcomes.

## 6. Key Points

1. *Fusobacterium nucleatum* density is significantly higher in UC patients unresponsive to anti-TNF therapy.
2. TNF-α mRNA expression is elevated in both responders and nonresponders compared to healthy controls.
3. *F. nucleatum* may contribute to treatment resistance by modulating intestinal inflammation and TNF-α expression.
4. Identifying *F. nucleatum* levels could help predict patient response to anti-TNF therapy in UC.

## Figures and Tables

**Table 1:** The TNF-α and reference gene expression primer sequences designed by our team.

*Fusobacterium nucleatum*	F: ATTTACACCATGTTGTCCTAATGC R: CCAGCAGGTAAAGCGAACC
TNF-α	F: CAGCCTCTTCTCCTTCCTGAT R: GCCAGAGGGCTGATTAGAGA
PCAT1	F: TGTGCCTCTAAGTGCCAGTGC R: GGTGATGTTGCGGTTTGTCTCC
RNU6	F: CTCGCTTCGGCAGCACATATAC R: AATGGAACGCTTCACGAATTTGC
GAPDH	F: TGACAACTTTGGTATCGTGGAAGG R: CCAGTAGAGGCAGGGATGATGTTC
	

**Table 2:** Demographics and disease characteristics of ulcerative colitis patients.

Primary response of anti-TNF-α therapy	Responder group (*n* = 10)	Nonresponder group (*n* = 10)	Control group (*n* = 10)	*p*
Mean age (year ± SD)	39.6	43.9	55.3	0.001*⁣*^*∗*^
Gender/male, *n* (%)	8 (80)	6 (60)	4 (40)	NS
Smoking status, *n* (%)				
Nonsmoker	5 (50)	5 (50)	4 (40)	NS
Ex-smoker	3 (30)	3 (30)	4 (40)	
Active smoker	2 (20)	2 (20)	2 (20)	
IBD in family history, *n* (%)	2 (20)	3 (30)	NA	NS
Comorbidities, *n* (%)	2 (20)	4 (40)	3 (30)	NS
Mean disease duration (year ± SD)	8.6 ± 4.5	9.1 ± 2.9	NA	NS
UC disease extention, *n* (%)				
E1:proctitis	0 (0)	0 (0)	NA	NS
E2:left-sided UC (distal to splenic flexure)	4 (40)	2 (40)		
E3:extensive UC (proximal to splenic flexure)	6 (60)	8 (60)		
UC disease activity, *n* (%)				
Endoscopic active disease (EMS^a^ ≥ 2)	10 (100)	10 (100)	NA	NS
Extraintestinal manifestations, *n* (%)	4 (40)	6 (60)	NA	NS
5-ASA therapy, *n* (%)				
Discontinued	0 (0)	0 (0)	NA	NS
Ongoing	9 (90)	10 (100)		
Intoleran/side effect	1 (10)	0 (0)		
IM therapy				
Discontinued	4 (40)	1 (10)	NA	NS
Ongoing	4 (40)	6 (60)		
Intoleran/side effect	2 (20)	3 (30)		
Biopsy location	Rectum	Rectum	Rectum	NA

Abbreviations: 5-ASA, 5-aminosalicylate; anti-TNF-α, antitumor necrosis factor α; EMS, endoscopic mayo subscores; IBD, inflammatory bowel disease; IM, Immunomodulator; NA, not applicable; NS, not significant; UC, ulcerative colitis.0.0050.0050.0050.005.

^a^[26].

⁣^*∗*^*p* < 0.005 was accepted to be statistically significant.

**Table 3:** *Fusobacterium nucleatum* density.

Cases	2^^dCT^	Fold change	Standard deviation	*p*-Value
Control	0.000472816	1.00	0.00	0.6088^a^ 0.0002^b^

Response	0.002032983	1.83	2.13	0.0028^c^

No response	0.072659607	5.02	3.24	—

^a^Control/*p*-value with response.

^b^Control/no response *p*-value.

^c^ *p* value with response/no response.

**Table 4:** TNF-α mRNA expression level.

Cases	2^ΔΔCT^	Fold change	Standard deviation	*p*-Value
Control	0.000147746	1	0	<0.0001^a^ <0.0001^b^

Response	0.019821124	6.51	1.39	0.5765^c^

No response	0.047144594	7.14	1.94	—

^a^Control/*p*-value with response.

^b^Control/no response *p*-value.

^c^ *p* value with response/no response.

## Data Availability

All relevant data analyzed during this study are included in this published article.
